# Prevalence of poppers use and its sexual risks among men who have sex with men in southwestern China: a cross-sectional study

**DOI:** 10.1186/s12889-018-6010-8

**Published:** 2018-09-10

**Authors:** Huailiang Chen, Yang Yang, Yuling Huang, Yingxue Dai, Jianxin Zhang

**Affiliations:** 1 Department of Infectious Diseases Prevention and Healthcare, the People’s Hospital of Chengdu Tianfu New Area, Chengdu, China; 2 Department of Health Care Chengdu Shuangliu District Maternal and Child Health Hospital, Chengdu, China; 3 Department of Sexually Transmitted Diseases Control and Prevention, Pidu District Center for Disease Control and Prevention, Chengdu, China; 4 Department of Infectious Diseases Control and Prevention, Chengdu Center for Disease Control and Prevention, Chengdu, China; 5 West China School of Public Health (West China Forth Hospital), Sichuan University, Sichuan Province, Chengdu, China

**Keywords:** Poppers use, Sexual risks, MSM, China

## Abstract

**Background:**

To investigate the prevalence of poppers use and its relationship with sexual risk behaviors among men who have sex with men (MSM) in southwestern China.

**Methods:**

This cross-sectional study was conducted in three cities of southwestern China between July and September 2016. Anonymous questionnaire survey was administered to collect data on demographics, drug use, sexual behaviors, history of STIs and HIV infection. Logistic regression analysis was performed to explore factors correlated with sexual risk behaviors including group sex and unprotected anal intercourse (UAI).

**Results:**

Of the 1122 participants included in the study, 24.1% reported a history of poppers use. 11.6% MSM reported ever engaging in group sex and 36.2% participants reported UAI with non-regular male partners in the past 12 months. Multivariate logistic analysis showed that age > 25 (OR = 2.96, 95% CI 1.87–4.68), seeking sex partners through the internet (OR = 3.16, 95% CI 1.59–6.29), preferring receptive anal intercourse (OR = 1.91, 95% CI 1.12–3.26) and ever using poppers (OR = 1.88, 95% CI 1.25–2.83) were positively associated with engaging in group sex. Lower levels of education (OR = 1.93, 95% CI 1.33–2.80) and ever using poppers (OR = 1.44, 95% CI 1.01–2.05) were significantly correlated with UAI with non-regular partners.

**Conclusions:**

The study suggested poppers was prevalent among MSM and its use was significantly associated with sexual risk behaviors. Given high prevalence of HIV among this subpopulation, comprehensive measures are needed to decrease poppers use and its potential risk for HIV transmission in southwestern China.

## Background

Recreational drug use among men who have sex with men (MSM) has been a worldwide public health concern in the past decades [1–4]. Differing from injection drug use (e.g., injecting heroin), which places users at a greater risk for HIV infection through sharing syringes, recreational drug users are at a higher risk for HIV acquisition that results from risky sexual behaviors, such as having more casual or commercial sex partners, unprotected anal intercourse (UAI) and engaging in group sex [5–7].

Prior studies from western countries reported that methamphetamine or ecstasy were the most commonly used drugs among MSM [8, 9], which was consistent with findings in earlier years of China [10, 11]. In recent years, several studies with large sample size approached the issue of drug use and found poppers (amyl nitrites) had outweighed methamphetamine or ecstasy and become the most popular recreational drug among MSM in coastal metropolises of China [12–14].

Findings from specific countries have demonstrated the associations between poppers use and sexual risk behaviors among samples of MSM [15, 16]. However, it is unknown whether these associations exist among MSM in China. Reports from coastal cities and online surveys in China have explored the prevalence and correlates of poppers use among MSM [12, 17] and a very small number of studies from north or east coastal cities of China explored the relationship between poppers use and sexual risk behaviors (having more sex partners and unprotected receptive anal intercourse) [18]. Still, more evidence is needed. This study was designed to explore the prevalence of poppers use, as well as its sexual risks among MSM in southwestern China, which could contribute to the existing literature. Additionally, understanding of these issues could further promote effective intervention about HIV/AIDS prevention among MSM in China.

## Methods

### Study design and participants

This cross-sectional study was conducted in three cities (Deyang, Xichang and Yibin) of Sichuan province during the period of July and September, 2016. Convenience sampling method was used to recruit subjects and MSM participants were invited by local community-based organizations (CBOs). Each CBO is operated by 2~ 3 full-time MSM staff who are trained and guided by the local Centers for Disease Control and Prevention (CDCs). CBO staff introduced the study to potential participants when providing voluntary counseling & testing (VCT) service for MSM and regularly reaching out in community (such as gay bars, public bathrooms and parks) to carry out health education activities. Recruited participants were encouraged to refer their MSM peers.

The inclusion criteria for participants were: male, ≥18 years, self-reported sexual experience with men and willing to provide informed consent. After confirming the eligibility of recruited participants, trained staff from CBOs fully explained the purpose, contents and procedures of the study to them. Verbal informed consent was obtained from MSM participants and then self-administered paper-and-pencil questionnaire survey was conducted anonymously at private rooms of local CBOs. Each participant was paid 25 CNY (~4 USD) for transportation fees.

### Data collection

The questionnaire survey took 10~ 20 min to complete and was used to collect socio-demographic characteristics (e.g., age, official residence location, education, occupation, marital status and monthly individual income), drug use behaviors, sexual behaviors, self-reported history of STIs and HIV infection from MSM participants.

#### Drug use behaviors

Participants were asked to report their lifetime use of rush poppers, capsule zero (5-MEO-DIPT, foxy), crystal meth, ecstasy, magu, ketamine, happy water (a mixture of crystal meth, ecstasy and ketamine), GHB, cannabis, bath salt (or cathinone), red crystal meth (extracts from methamphetamine) and heroine. Participants were asked to specify the drug’s name in case it was not listed.

#### Sexual Behaviors and STIs/HIV Infection.

Participants were asked about their age at sexual debut, sexual orientation (homosexual/heterosexual/bisexual/unknown), preferred sexual role with males (insertive/receptive/versatile), routes to seek sex partners (internet/bar/dance halls/ park/public bath) and number of sex partners in the past 12 months. They were also asked whether they had regular (spouse, boy/girlfriend, lover) or non-regular (commercial/ casual/ anonymous) sex partners and their status of condom use (never/sometimes/every time) in the past 12 months. Condom use variables were re-coded as unprotected (never or sometimes) versus protected (every time). Furthermore, MSM participants were asked whether they had engaged in group sex. Other variables included history of STI/HIV test (yes vs. no) and results of STI/HIV test (positive vs. negative).

### Data analysis

Data were entered by Epi Data software 3.1 (The Epi Data Association, Odense, Denmark) and analyzed by SPSS 16.0 (SPSS, Inc., Chicago, IL, USA). Continuous variables and category variables were expressed as means and proportions, respectively. Chi-square tests were conducted to compare the differences in socio-demographics, sexual behaviors and HIV/STI infection by poppers use status. Stepwise logistic regression analysis was used to evaluate associated factors for UAI with non-regular partners and engaging in group sex. Variables with *P* < 0.2 level in the univariate analysis were selected for multivariate logistic regression analysis and variables with *P* < 0.05 in the multivariate logistic regression analysis were retained in the final model.

## Results

### Characteristics of participants

A total of 1167 MSM were invited to participate in this study. After excluding MSM who were < 18 years (1.1%) and those who were not willing to report their drug use status (2.7%), 1122 participants were included in the final analysis. The mean age of enrolled MSM was 30.0 years (SD = 9.0). Of the 1122 eligible participants, almost all (98.2%) officially resided in Sichuan province and over two-fifths (44.1%) had obtained at least a college education level. Most (70.5%) of MSM participants were single. More than half (52.6%) reported to be solo business owners or service providers and three-fifths (60.0%) reported a monthly individual income of 3000 Yuan or more. Overall, 128 (11.6%) MSM reported ever engaging in group sex and more than one-third (36.2%) reported having UAI with non-regular partners in the past 12 months. 142 (12.7%) MSM reported being HIV-positive and 149 (13.3%) MSM reported a history of STIs.

Among the participants, 311 (27.7%) participants reported using at least one type of recreational drug in their lifetime, of whom 36.0% were polydrug users. Poppers (24.1%) were the most commonly used recreational drug, followed by methamphetamine (7.1%), zero capsule (6.1%), magu (2.0%), ecstasy (0.4%), ketamine (0.4%) and happy water (0.2%). Comparisons of socio-demographic characteristics, sexual behaviors, history of STIs and HIV infection between poppers users and non-users were presented in Table 1. Compared to non-users, poppers users tend to be younger and single. They were more likely to seek sexual partners through the internet, prefer receptive or versatile anal intercourse, practice more UAI with regular sexual partners in the past 12 months, engaged in group sex more frequently and report higher prevalence of STIs.

**Table 1:** Comparison of characteristics among MSM by poppers using status (*n* = 1122)

Characteristics	Total (*n*,%)	Popper users (*n*,%)	Non-users (*n*,%)	*P* value
Age (years), mean ± SD	30.0 ± 9.0	27.9 ± 6.6	30.6 ± 9.5	
≤25	418(37.3)	104(38.5)	314(36.8)	< 0.001
26–35	428(38.1)	124(45.9)	304(35.7)	
> 35	276(24.6)	42(15.6)	234(27.5)	
Residence in Sichuan Province				
Yes	1091(98.2)	262(97.8)	829(98.3)	0.597
No	20(1.8)	6(2.2)	14(1.7)	
Education				
Senior high school and below	622(55.9)	147(54.6)	475(56.3)	0.639
College and above	491(44.1)	122(45.4)	369(43.7)	
Occupation				
Enterprise, public institution or government	263(23.6)	71(26.3)	192(22.8)	0.110
Service industry, solo business owner	585(52.6)	147(54.4)	438(51.9)	
Retired, unemployed or student	265(23.8)	52(19.3)	213(25.3)	
Marital status				
Never married	788(70.5)	208(77.3)	580(68.4)	0.013
Married or cohabitating	248(22.2)	49(18.2)	199(23.5)	
Divorced or widowed	81(7.3)	12(4.5)	69(8.1)	
Individual monthly income (Yuan)				
< 3000	398(40.0)	102(38.6)	296(40.5)	0.609
≥3000	597(60.0)	162(61.4)	435(59.5)	
Sexual orientation				
Homosexual	796(71.5)	189(70.0)	607(71.9)	0.616
Bisexual	301(27.0)	78(28.9)	223(26.4)	
Uncertain	17(1.5)	3(1.1)	14(1.7)	
Age at sexual debut (years)				
< 18	308(27.7)	98(36.3)	210(24.9)	< 0.001
≥18	805(72.3)	172(63.7)	633(75.1)	
Seeking sex partners through the internet				
Yes	884(79.1)	226(84.0)	658(77.6)	0.025
No	233(20.9)	43(16.0)	190(22.4)	
Preferred sexual role with males				
Insertive anal intercourse	281(25.3)	39(14.4)	242(28.8)	< 0.001
Receptive anal intercourse	290(26.1)	89(33.0)	201(23.9)	
Versatile anal intercourse	540(48.6)	142(52.6)	398(47.3)	
UAI with regular partners in the past 12 months				
Yes	248(55.7)	94(70.7)	154(49.4)	< 0.001
No	197(44.3)	39(29.3)	158(50.6)	
UAI with non-regular partners in the past 12 months				
Yes	312(36.2)	88(41.5)	224(34.5)	0.064
No	550(63.8)	124(58.5)	426(65.5)	
Number of sex partners in the past 12 months				
≤1	227(20.2)	52(19.3)	175(20.5)	0.728
≥2	895(79.8)	218(80.7)	677(79.5)	
Group sex				
Yes	128(11.6)	46(17.3)	82(9.7)	0.001
No	980(88.4)	220(82.7)	760(90.3)	
Self-reported HIV infection				
Yes	142(12.7)	41(15.2)	101(11.9)	0.172
No	980(87.3)	229(84.8)	751(88.1)	
Self-reported gonorrhoea, condyloma acuminata or syphilis infection				
Yes	149(13.3)	50(18.5)	99(11.7)	0.005
No	968(86.7)	220(81.5)	748(88.3)	

### Factors associated with engaging in group sex

Results exploring factors associated with engaging in group sex are summarized in Table 2. Univariate analysis indicated the following variables were positively associated with engaging in group sex (*P* < 0.2): age > 25 years old, being bisexual, seeking sex partners through the internet, preferring receptive anal intercourse and ever using poppers. These factors were further included in multivariate analysis and the following variables were retained in the final multivariate model (*P* < 0.05): age > 25 (OR = 2.96, 95% CI 1.87–4.68) (versus age ≤ 25), seeking sex partners through the internet (OR = 3.16, 95% CI 1.59–6.29), preferring receptive anal intercourse (OR = 1.91, 95% CI 1.12–3.26) (versus preferring insertive anal intercourse) and ever using poppers (OR = 1.88, 95% CI 1.25–2.83).

**Table 2:** Factors correlated with engaging in group sex (*n* = 1122)

Factors	Univariate analysis		Multivariate analysis	
	OR(95% CI)	*P* value	aOR(95% CI)	*P* value
Age (years)				
≤25	1			
> 25	2.08(1.35–3.19)	0.001	2.96(1.87–4.68)	< 0.001
Education				
Senior high school and below	1			
College and above	1.05(0.73–1.52)	0.792		
Occupation				
Enterprise, public institution or government	1			
Service industry, solo business owners and others	0.88(0.58–1.35)	0.561		
Marital status				
Single	1			
Married or cohabitating	0.78(0.49–1.25)	0.302		
Divorced or widowed	0.90(0.43–1.85)	0.770		
Monthly individual income, Yuan				
< 3000	1			
≥3000	0.83(0.57–1.21)	0.330		
Sexual orientation				
Homosexual	1			
Bisexual	0.58(0.37–0.93)	0.023	0.65(0.39–1.07)	0.087
Uncertain	0.41(0.05–3.16)	0.395	0.57(0.07–4.52)	0.598
Seeking sex partners through the internet				
No	1			
Yes	3.43(1.77–6.66)	< 0.001	3.16(1.59–6.29)	0.001
Preferred sexual role with males				
Insertive anal intercourse	1			
Receptive anal intercourse	1.66(1.01–2.73)	0.046	1.91(1.12–3.26)	0.017
Versatile anal intercourse	0.92(0.57–1.48)	0.723	1.08(0.66–1.78)	0.755
Age at sexual debut (years)				
≥18	1			
< 18	0.27(0.83–1.96)	0.272		
Ever using poppers				
No	1			
Yes	1.94(1.31–2.87)	0.001	1.88(1.25–2.83)	0.002

*CI* confidence interval, *OR* odds ratio, *aOR* adjusted odds ratio

### Factors associated with UAI with non-regular partners

Table 3 demonstrated factors correlated with UAI with non-regular partners in the past 12 months. Univariate analysis revealed that age ≤ 25 years old, having less than senior high school education, working in service industry, being solo business owners or others, monthly individual income< 3000 Yuan, being uncertain about sexual orientation, seeking sex partners through the internet, preferring receptive or versatile anal intercourse, age at sexual debut< 18 years and ever using poppers were significantly associated with UAI with non-regular partners (*P* < 0.2). In the final multivariate logistic regression model, UAI with non-regular partners were significantly correlated with the following factors (*P* < 0.05): having less than senior high school education (OR = 1.93, 95% CI 1.33–2.80) and ever using poppers (OR = 1.44, 95% CI 1.01–2.05).

**Table 3:** Factors correlated with unprotected sex with non-regular partners in the past 12 months (*n* = 1122)

Factors	Univariate analysis		Multivariate analysis	
	OR(95% CI)	*P* value	aOR(95% CI)	*P* value
Age (years)				
> 25	1			
≤25	1.65(1.24–2.19)	0.001	1.17(0.77–1.78)	0.464
Education				
College and above	1			
Senior high school and below	2.19(1.64–2.93)	< 0.001	1.93(1.33–2.80)	0.001
Occupation				
Enterprise, public institution or government	1			
Service industry, solo business owners and others	1.71(1.21–2.42)	0.002	0.92(0.60–1.43)	0.717
Marital status				
Single	1			
Married or cohabitating	0.98(0.70–1.37)	0.912		
Divorced or widowed	1.00(0.57–1.76)	0.993		
Monthly individual income (Yuan)				
≥3000	1			
< 3000	1.75(1.29–2.38)	< 0.001	1.40(0.97–2.03)	0.073
Sexual orientation				
Homosexual	1			
Bisexual	0.98(0.71–1.34)	0.874	1.05(0.72–1.53)	0.811
Uncertain	2.68(0.75–9.59)	0.130	1.92(0.48–7.65)	0.352
Seeking sex partners through the internet				
No	1			
Yes	0.59(0.43–0.83)	0.002	0.77(0.50–1.20)	0.247
Preferred sexual role with males				
Insertive anal intercourse	1			
Receptive anal intercourse	1.43(0.95–2.13)	0.084	0.92(0.57–1.49)	0.743
Versatile anal intercourse	1.43(1.00–2.04)	0.050	1.24(0.83–1.85)	0.295
Age at sexual debut (years)				
≥18	1			
< 18	1.74(1.28–2.36)	< 0.001	1.28(0.84–1.93)	0.249
Ever using poppers				
No	1			
Yes	1.35(0.98–1.85)	0.064	1.44(1.01–2.05)	0.042

*CI* confidence interval *OR*, odds ratio aOR, adjusted odds ratio

## Discussion

Consistent with results from the coastal metropolises [12–14], the current study found poppers was the most prevalent recreational drug among MSM in southwestern China. Of the 1122 participants, 270 (24.1%) reported using poppers in their life time, which was higher than reports in Shenyang (19.2%) [13] and Changsha (16.1%) [14], but lower than the 3-month use rate (26.8%) among MSM in Beijing [17]. There might be several reasons contributing to the popularity of poppers. For one thing, as most recreational drugs did, poppers use was reported to prolong duration of anal intercourse and enhance sexual experience [12, 19]. For another, unlike other recreational drugs (such as methamphetamine, ecstasy and ketamine), poppers have not been included in the list of illegal drugs and could be easily accessed through the internet in China. Besides, we found poppers users were more likely to be younger and unmarried, seek sexual partners through the internet and prefer receptive/versatile anal intercourse. It suggests increased attention should be given to the issue of poppers use and regular surveillance system needs to be established to monitor the dynamics of poppers use among these subgroups of MSM.

In the present study, 11.6% MSM reported having ever participated in group sex, which was comparable with related reports in China [20, 21], but lower than reports in western countries [16, 22]. Only a few research studies in China explored the correlates of group sex, while studies assessing the association between poppers use and group sex events are rarely found. In the multivariate model, we found poppers use was significantly associated with group sex, which was in accordance with findings from the US [23]. Poppers use was reported to enhance sexual function and promote sexual pleasure [18, 24], and thus MSM might use poppers to maximize the experience of group sex.

Moreover, older MSM were more likely to report participating in group sex, and similar result was indicated among MSM in Australia [22]. MSM with older age may represent a subgroup of participants who were more adventurous in sexual behaviors and active in sexual sensation seeking [25]. Meanwhile, seeking sex partners through the internet were positively associated with group sex. The rapid development of internet provides MSM with much convenience in seeking sex partners and they may organize or participate in a sex party via internet with ease. The fact that most (79.1%) of the participants in this study reported seeking sex partners through the internet might partly support the view. Other indicator, such as preferring receptive anal intercourse, suggests that specific sexual role in anal intercourse could be associated with the tendency to engage in group sex. Men who preferred receptive anal intercourse are more vulnerable to HIV infection during anal intercourse [26] and group sex was reported to be associated with unprotected receptive anal intercourse [16]. Thus, those who preferred receptive anal intercourse were exposed to elevated risk of HIV acquisition when engaging in group sex and close attention should be paid to this subpopulation.

In addition, poppers use was found to be significantly correlated with UAI with non-regular sex partners in the past 12 months. Like other recreational drugs, poppers may also have the neurological effects on sexual behaviors such as heightening libido and reducing sexual inhibition [24]. Thus, MSM were more likely to seek casual sex partners and practice higher levels of UAI after drug use. Similar to the findings in other studies [27], participants with lower levels of education were more likely to report UAI with non-regular sex partners, probably because they have limited access to knowledge about HIV/AIDS.

There are a few potential limitations to our study. First, although MSM participants were recruited from different venues, still selection bias may exist, as there was no sampling frame and non-probabilistic sampling method was used. Second, the results were based on self-administered questionnaire survey, which may result in recall bias. Third, data on drug use and sexual behaviors are still sensitive in China, and thus social desirability bias and concealment of information are possible. However, the purpose and contents of the study were fully explained to participants and confidentiality of their information was guaranteed by self-administered anonymous questionnaire survey in private rooms, which could possibly minimize the bias. Fourth, the causal relationship between associated factors and risky sexual behaviors was weak, as data were collected from a cross-sectional study. Finally, this is an exploratory study and the variable selections in the multivariable models were solely based on *P*-values. This could cause a problem that some predictor variables may not meet the criterion of biological plausibility. For example, we are unable to explain why men who preferred receptive anal intercourse was more likely to be engaged in group sex and further studies are needed to figure it out.

## Conclusions

Our study indicated recreational drugs, especially poppers, were prevalent among MSM and use of poppers was significantly associated with sexual risk behaviors including group sex and UAI. Given high prevalence of HIV among MSM, popularity of poppers use may further fuel the epidemic within this subpopulation. Thus, Close attention should be given to the dynamics of poppers use and interventions targeting to both poppers use and sexual risk behaviors are needed to prevent the spread of HIV transmission in China.

## Abbreviations

CBOs: Community-based organizations
CDCs: Centers for disease control and prevention
CI: Confidence -interval
HIV: Human immunodeficiency virus
MSM: Men who have sex with men
OR: Odds ratio
SD: Standard deviation
STIs: Sexually transmitted infections
UAI: Unprotected anal intercourse
VCT: Voluntary counseling & testing
